# Circulating exosomal small RNAs are promising non‐invasive diagnostic biomarkers for gastric cancer

**DOI:** 10.1111/jcmm.16077

**Published:** 2020-11-09

**Authors:** Lichen Ge, Nan Zhang, Dandan Li, Yingmin Wu, Hongsheng Wang, Junjun Wang

**Affiliations:** ^1^ Department of Clinical Laboratory Jinling Hospital Medical School of Nanjing University Nanjing China; ^2^ Key Laboratory of Chiral Molecule and Drug Discovery School of Pharmaceutical Sciences Sun Yat‐sen University Guangzhou China

**Keywords:** diagnosis, gastric cancer, non‐coding RNA, PIWI‐interacting RNAs, serum exosomes

## Abstract

Dysregulation of small non‐coding RNA (ncRNA) is associated with various human diseases including cancer. This study aimed to evaluate the circulating exosomal small RNAs including microRNAs (miRNAs) and P‑element‑induced wimpy testis (PIWI)‐interacting RNAs (piRNAs) as sensitive and specific non‐invasive biomarkers for gastric cancer (GC) diagnosis. Serum exosomal small RNA transcriptome was examined using unique molecular identifiers (UMI) small RNA sequencing. Dysregulated miRNAs and piRNAs were verified in 70 GC patients and 60 healthy controls (HC) by reverse transcription quantitative PCR. The expressions of miR‐1307‐3p, piR‐019308, piR‐004918 and piR‐018569 in serum exosomes were significantly increased in GC group as compared to those in HC group. Moreover, GC patients with metastasis had significantly higher expression levels of piR‐004918 and piR‐019308 than GC patients without metastasis. The area under the curve (AUC) for miR‐1307‐3p, piR‐019308, piR‐004918 and piR‐018569 in the GC group was 0.845, 0.820, 0.754 and 0.732, respectively. The combination of carcinoembryonic antigen (CEA) and carbohydrate antigen (CA) 199 can improve the AUC of miR‐1307‐3p to 0.902 and piR‐019308 to 0.914 for GC diagnosis. In conclusion, our findings indicate that serum exosomal piRNAs are promising non‐invasive diagnostic biomarkers for GC patients and potential markers for monitoring metastasis.

## 1 INTRODUCTION

Gastric cancer (GC) is the fifth most common cancer worldwide and the third leading cause of death from malignant tumours.^1^ In China, the number of people who were newly diagnosed with GC was approximately 679,100 and almost 498,000 people died in 2015.^2^ As most newly diagnosed with GC are identified at an advanced stage, five‐year survival rate of patients is low.^3^ The most common‐used GC tumour markers, including carcinoembryonic antigen (CEA), carbohydrate antigen (CA) 199, CA242 and CA724, are not sensitive or specific enough for GC screening and diagnosis, particularly for the early GC patients.^4^, ^5^ Considering that the five‐year survival rate of patients with early GC can reach to 90%,^6^ there is an urgent need to develop more effective and simple diagnostic biomarkers for detection of early GC.

microRNAs (miRNAs) are a class of small non‐coding RNA (ncRNA) of 18 ~ 24 nucleotides (nt) in length that post‐transcriptionally regulate gene expression by binding to the 3’‐untranslated regions (3’‐UTRs) of target mRNA, leading its translational inhibition or degradation.^7^ In addition to miRNAs, P‑element‑induced wimpy testis (PIWI)‐interacting RNAs (piRNAs) are another novel class of small ncRNA, with a length of 26 ~ 32 nt, which was initially discovered in germ cells.^8^ The biogenesis of piRNAs could be controlled by PIWI proteins (PIWIL1, PIWIL2, PIWIL3 and PIWIL4), which are a subclass of the conserved Argonaute family of proteins.^9^ piRNAs could be more resistant to oxidation and degradation than miRNAs due to its 2’‐O‐methyl (2’‐O‐Me) modification on 3’ terminal base.^10^ Similar to miRNAs, piRNAs post‐transcriptionally regulate gene expression in cytoplasm.^11^ The number of piRNAs has been identified more than 30,000 in human genome, which is much greater than that of miRNAs (~2000).^12^

Dysregulation of miRNAs and piRNAs expressions might be associated with various human diseases especially carcinogenesis by regulating tumour suppressor genes or oncogenes.^12^, ^13^, ^14^ Accumulating studies reveal circulating miRNAs might be useful diagnostic and prognostic biomarker for various cancer.^15^ Serum piR‐020619 and piR‐020450 are promising novel biomarkers for early detection of colorectal cancer.^16^ Elevating piR‐823 level could suppress the growth of GC cells.^17^ Another study showed that a three‐piRNA cluster associated with recurrence‐free survival (RFS) of patients with gastric adenocarcinoma.^18^ In light of these studies, circulating piRNAs might be novel potential non‐invasive biomarkers for GC diagnosis.

Extracellular vesicles (EVs) are type of membrane particle released from nearly almost cell types and could been found in various biofluids, including serum, plasma, urine, saliva and cerebrospinal fluid.^19^, ^20^ EVs of endosomal origin are called exosomes with the diameter range between 30 ~ 150 nm, which contain various functional proteins, mRNA, ncRNA, DNA and metabolites originated from cell.^20^, ^21^ Accumulating evidence suggests circulating exosomes in biofluids and tumour microenvironment might be involved in various cancer development and progression.^22^, ^23^, ^24^, ^25^ Exosomes isolated from biofluids of cancer patients have been shown to contain tumour‐derived functional molecules, which might be powerful non‐invasive diagnostic and prognostic tool for cancer.^21^, ^26^ Serum exosomal long non‐coding RNA HOTTIP was significantly higher in GC patients than in healthy individuals, which suggested that HOTTIP is a potential novel diagnostic biomarker for GC.^27^ Plasma exosomal miR‐23b could be a biomarker for prediction of recurrence and progression of GC patients.^28^ However, study of serum exosomal small ncRNA as biomarker for GC is limited.

In this study, we investigated the expressions of miRNAs and piRNAs in serum exosomes of GC patients and healthy controls (HC) to evaluate its potential as diagnostic biomarkers for GC. Our data showed that in addition to miR‐1307‐3p, serum exosomal piR‐019308, piR‐004918 and piR‐018569 are promising non‐invasive diagnostic biomarkers for GC patients. Moreover, serum exosomal piR‐019308 and piR‐004918 are potential markers for monitoring metastasis.

## 2 MATERIALS AND METHODS

### 2.1 Sample collection and study design

Serum samples from 70 GC patients and 60 HC who had no history of basic or chronic diseases were collected from Jinling Hospital of Nanjing University using gel barrier tubes. Samples were stored at −80°C until exosomal RNA isolation. All GC patients were diagnosed on the basis of the histopathology by biopsy or endoscopic examination, and serum samples were collected at the time of diagnosis before surgery or radiochemotherapy. Informed consent was obtained for all individuals. Ethics approval was obtained from the Ethics Committee of Jinling Hospital of Nanjing University. The demographic characteristics of participating patients and the HC are described in Table S1.

### 2.2 Exosomes isolation

Frozen serum samples were incubated at 37ºC in a water bath until samples were completely thawed. The thawed samples were excluded cellular material, including thrombocyte fragments and particles larger than 0.8 µm using syringe filters (0.8 µm filter pore size, Millipore). Then removing some of the larger vesicles by passing through 0.22 µm filter (Millipore). Exosomes were isolated from pre‐filtered serum by exoRNeasy Serum/Plasma Kits (Qiagen).

### 2.3 Nanoparticle tracking analysis

Serum exosomes eluted in 100 µL Buffer XE using exoRNeasy Serum/Plasma Kits were diluted in PBS to a final volume of 1 ml. The size distribution and concentration of exosomes were analysed by nanoparticle tracking analysis using ZetaView particle tracker (Particle Metrix). Ideal measurement concentrations were found by pre‐testing the ideal particle per frame value (140 ~ 200 particles/frame). The manufacturer's default software settings for exosomes were selected accordingly. Measurement data from the ZetaView were analysed using the corresponding software (ZetaView 8.02.28).

### 2.4 Transmission electron microscopy

Freshly isolated exosomes suspensions were fixed with 4% paraformaldehyde in 0.1 M phosphate buffer (pH 7.4). A drop of each sample was placed on a carbon‐coated copper grid and negative staining with 1% uranyl acetate for 1 minute. The preparations were examined with a transmission electron microscope (JEM‐1400EX, JEOL Ltd., Japan) at an acceleration voltage of 120 kV.

### 2.5 Western blot analysis

Serum exosomes eluted in 100 µL Buffer XE were concentrated to 10 µL using ultrafiltration (100 KDa pore size, Millipore). Concentrated exosomes were lysed with 40 µL of RIPA buffer. Then, 10 µg of total protein were prepared for Western blot analysis performed as previously described.^29^ The antibodies used in this study were specific for TSG101 (Proteintech, 14497‐1‐AP, 1:1000), CD9 (Abcam, ab92726, 1:1000) and Alix (Proteintech, 12422‐1‐AP, 1:1000).

### 2.6 Exosomal RNA extraction

Exosomal RNA was isolated by exoRNeasy Serum/Plasma Kits (Qiagen) according to the manufacturer's instructions.^30^ Briefly, 800 μL pre‐filtered serum was mixed with equal volume buffer XBP. The sample/XBP mix was added onto the exoEasy spin column and spin the device for 1 minute at 500 × g. Buffer XWP was added onto the exoEasy column and spun 5 min at 5000 × g. 700 µl of QIAzol was added onto the membrane of exoEasy column, spun for 5 min at 5000 × g to collect the lysate, then 1 µl *Caenorhabditis elegans* miR‐39 (cel‐39, 1 μM) were added into the lysate for further external control. Subsequently, 90 µl of chloroform was used for phase separation. The upper aqueous phase/ethanol mix, buffer RWT and buffer RPE were added into RNeasy MinElute spin column in sequence as specified in the manufacturer's instructions. Finally, RNA was eluted in 20 µl RNase‐free water. RNA integrity and size distributions were evaluated by the Agilent 2100 Bioanalyzer Instrument.

### 2.7 Unique Molecular Identifier (UMI) small RNA sequencing

Considering the quantity of RNA extracted from serum exosomes was extremely low (usually less than 200 ng per 1 mL serum),^31^ small RNA sequencing were performed using adapters containing unique molecular identifiers (UMI) to label each molecule before library construction. With UMI, library preparation from low quantity of starting material is allowed, the number of PCR amplification cycles is unlimited, the abundance of transcript expression is truly reflected, so that achieving accurate and unbiased quantification, which is more suitable for rare and precious clinical samples.^32^

Library was prepared with 6.5 μL total RNA for each sample (from four HC individuals and four GC patients). Total RNA was purified by electrophoretic separation on a 15% urea denaturing polyacrylamide gel electrophoresis (PAGE) gel, and small RNA regions corresponding to the 18 ~ 30 nt bands in the marker lane (14 ~ 30 ssRNA Ladder Marker, Takara) were excised and recovered. Then, the 18 ~ 30 nt small RNAs were ligated to adenylated 3’ adapters annealed to UMI, followed by the ligation of 5’adapters. The adapter‐ligated small RNAs were subsequently transcribed into cDNA by SuperScript II Reverse Transcriptase (Invitrogen) and then several rounds of PCR amplification with PCR Primer Cocktail and PCR Mix were performed to enrich the cDNA fragments. The PCR products were selected by agarose gel electrophoresis with target fragments 110 ~ 130 bp and then purified by QIAquick Gel Extraction Kit (Qiagen). The library was quality and quantitated in two methods: check the distribution of the fragments size using the Agilent 2100 bioanalyzer and quantify the library using real‐time quantitative PCR (QPCR) (TaqMan Probe). The final ligation PCR products were sequenced using the BGISEQ‐500 platform (BGI, China).

### 2.8 Sequencing data processing and differential small RNA analyses

The raw tags were processed to remove the tags with low quality, with 5’ contamination, without 3 primer, without insertion, with poly A, shorter than 18 nt. After filtering, the clean tags were mapped against miRBase for miRNA,piRNABank (http://pirnabank.ibab.ac.in) for piRNA with Bowtie 2. The small RNA expression level was calculated by counting absolute numbers of molecules using UMI. Differential expression analysis was performed using the PossionDis, FDR ≤ 0.001 and the absolute value of Log2Ratio ≥ 1 as the default threshold to judge the significance of expression difference.

### 2.9 Quantitative real‐time PCR

Briefly, 4 μL of RNA extracted from serum exosomes was reverse transcribed to cDNA using the miRcute Plus miRNA First‐Strand cDNA Kit (TIANGEN). Quantitative real‐time PCR using TB Green™ Premix Ex Taq™ II (Takara) was performed on a CFX96 Touch™ Real‐Time PCR Detection System (Bio‐Rad). The cycle parameters were as follows: 95°C for 15 min followed by 40 cycles of 94°C for 20 seconds, 60°C for 34 s and 65°C for 5 seconds. We calculated ΔCq by subtracting the Cq value of the exogenous reference cel‐39 from the raw Cq value. The expressions of targeted genes in each sample were normalized to the cel‐39. The absolute expression levels were calculated by the 2^−ΔΔCt^ method. The universal reverse primer and forward primer of cel‐39 were commercial reagent (TIANGEN), and other forward primers of targeted miRNAs and piRNAs were listed in Table S2.

### 2.10 Statistical analysis

Statistical analysis was performed with IBM SPSS Statistics 25.0, GraphPad Prism version 7.0, and MedCalc version 17.1. Between group differences were analysed by parametric two‐tailed unpaired Student's *t* tests for normally distributed data. Otherwise, the data were analysed by nonparametric Mann‐Whitney tests. A *P* value < .05 was considered statistically significant.

## 3 RESULTS

### 3.1 Identification of serum exosomes and characterization of serum exosomal RNA

**Figure 1:**
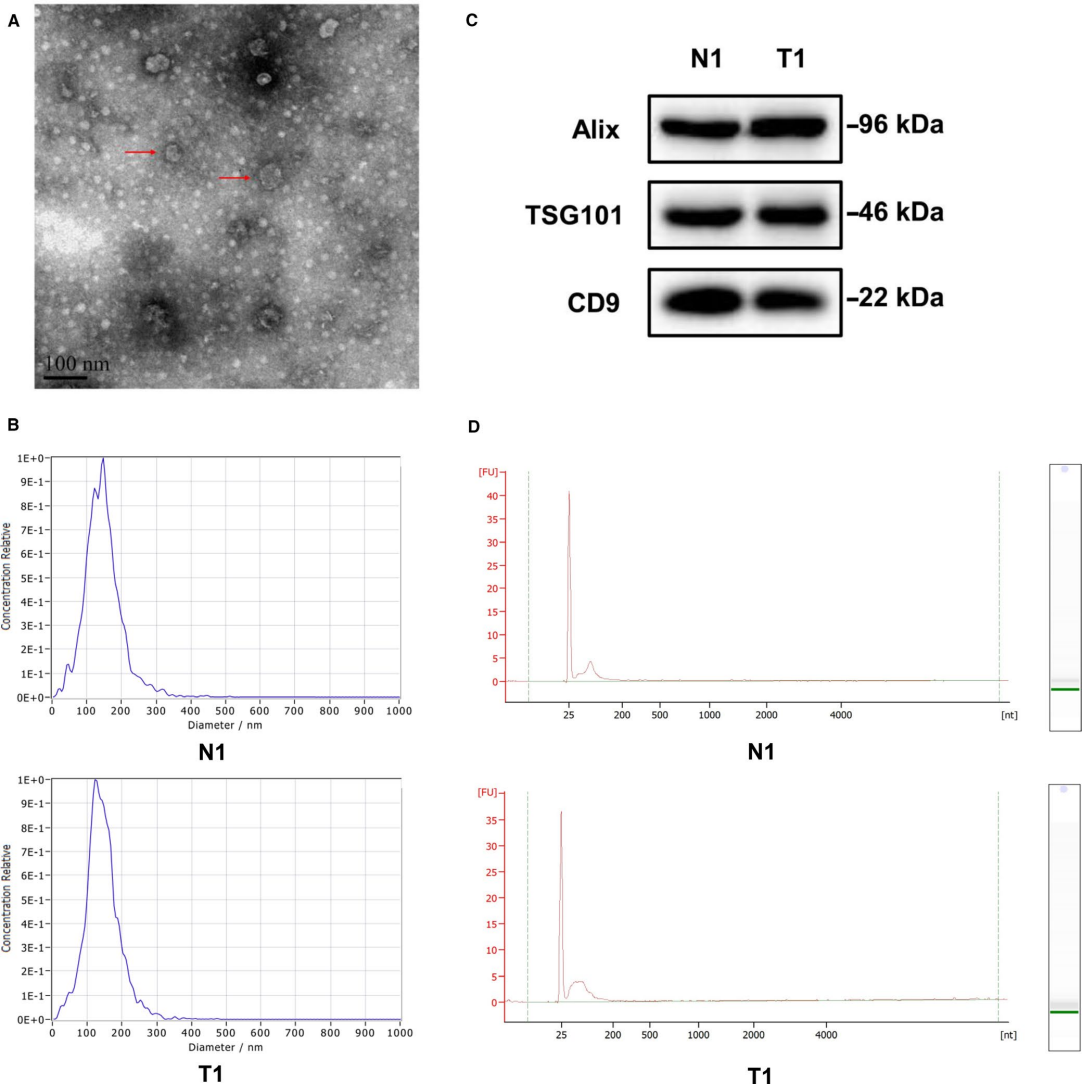
Identification of serum exosomes and characterization of serum exosomal RNA. A, Isolated particles were analysed using transmission electron microscopy which showed the typical size and morphology. Typical exosomes were highlighted using red arrows (scale bar = 100 nm); B, The sizes of isolated particles from 1 HC individual (N1) and 1 GC patients (T1) were characterized via the nanoparticle tracking analysis which revealed the majority of vesicle particles were mainly between 60 and 150 nm in diameter; C, Exosomes‐enriched protein markers including CD9, TSG101 and Alix were analysed by Western blotting in the isolated particles from 1 HC individual (N1) and 1 GC patients (T1); D, The RNA size distributions of serum exosomal RNA from 1 HC individual (N1) and 1 GC patient (T1) were analysed by use of the Agilent 2100 Bioanalyzer Instrument

Transmission electron microscopy showed the isolated particles have a typical size and morphology of exosomes (Figure 1A). Nanoparticle tracking analysis revealed that the particles were approximately 100 nm in diameter, which was consistent with the typical size of exosomes (Figure 1B). We further verified the exosomal surface marker proteins. Expression of CD9, TSG101 and Alix could be detected in the particles isolated from both HC and GC samples (Figure 1C). These data indicated that serum exosomes were successfully isolated.

The integrity and RNA size distributions of serum exosomal RNA were analysed by use of the Agilent 2100 Bioanalyzer Instrument. Results showed that the RNA integrity number (RIN) of almost all serum exosomal RNA samples were below 2.0, meanwhile, exosomal RNA samples from serum contained high proportions of small RNAs, which was consistent with the characterization of exosomal RNA (Figure 1D). These results indicated that the isolated exosomal RNA was suitable for further studies.

### 3.2 Profiling of serum exosomal miRNAs and piRNAs by UMI small RNA sequencing

**Figure 2:**
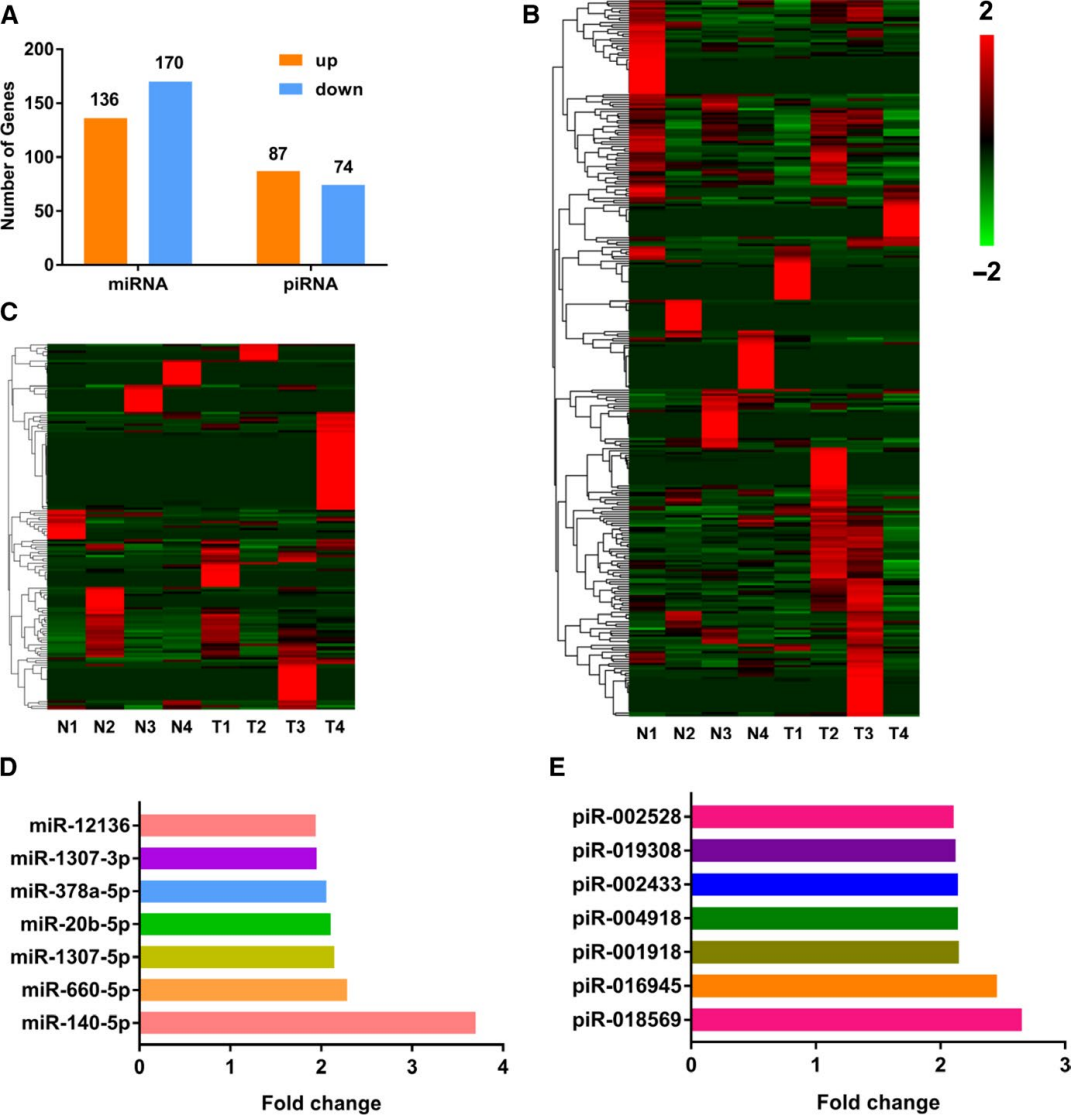
Statistic of detected miRNAs and piRNAs in UMI small RNA sequencing. A, Different miRNAs (including 136 up‐regulation and 170 down‐regulation in GC group compared with HC group) and piRNAs (including 87 up‐regulation and 74 down‐regulation in GC group compared with HC group) were found to be present in serum exosomes for UMI small RNA sequencing; (B‐C) Heatmaps of miRNAs (B) and piRNAs (C) that were differentially expressed in serum exosomes of 4 HC individuals (N1‐N4) and 4 GC patients (T1‐T4). Red represents high expression, and green represents low expression; (D‐E) Up‐regulated miRNAs (D) and piRNAs (E) in serum exosomes of 4 GC patients compared with 4 HC individuals according to the screening criteria

Libraries established from the serum exosomal RNA samples of 4 GC patients and 4 HC individuals were subjected to UMI small RNA sequencing. The sequence data showed 306 different miRNAs and 161 different piRNAs were found to be present in serum exosomes (Figure 2A). Heatmaps showed differential expression profiles of serum exosomal miRNAs (Figure 2B) and piRNAs (Figure 2C) between GC group and HC group. We chose the differentially expressed miRNAs and piRNAs as potential biomarkers for GC according to the following criteria: 1) fold change of up‐regulated miRNAs and piRNAs ranked top 10 respectively between the GC group and the HC group (Table S3); 2) no less than 15 transcripts parts per million (TPM) in at least two different libraries in GC group. As a result, 7 miRNAs (Figure 2D) and 7 piRNAs (Figure 2E) were selected for further evaluation in the training phase of the study.

### 3.3 The training phase of the study

**Figure 3:**
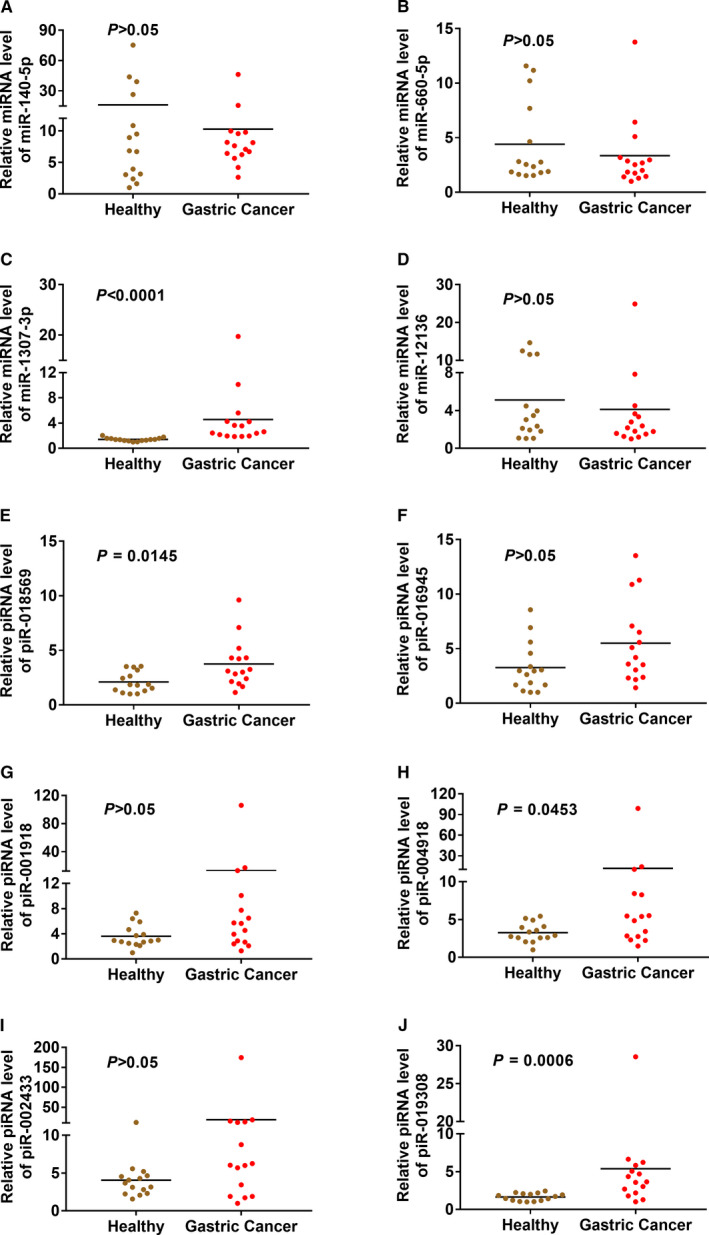
The expressions of candidate miRNAs and piRNAs in the training cohorts. The expressions of miR‐140‐5p (A), miR‐660‐5p (B), miR‐1307‐3p (C), miR‐12136 (D), piR‐018569 (E), piR‐016945 (F), piR‐001918 (G), piR‐004918 (H), piR‐002433 (I) and piR‐019308 (J) were evaluated by RT‐qPCR in serum exosomal RNA samples from 15 GC patients and 15 HC individuals in the training cohorts

The expressions of the 7 candidate miRNAs and 7 candidate piRNAs were evaluated by RT‐qPCR in serum exosomal RNA samples from 15 GC patients and 15 HC individuals in the training phase. Firstly, we excluded 3 candidate miRNAs (miR‐1307‐5p, miR‐20b‐5p, miR‐378a‐5p) and 1 candidate piRNA (piR‐002528) because Cq values of these candidates were greater than 32 in majority of samples. Among the remaining candidates, the expression of miR‐1307‐3p and three piRNAs (piR‐018569, piR‐004918, piR‐019308) was significantly up‐regulated in GC group compared with HC group (Figure 3).

### 3.4 The validation phase of the study

**Figure 4:**
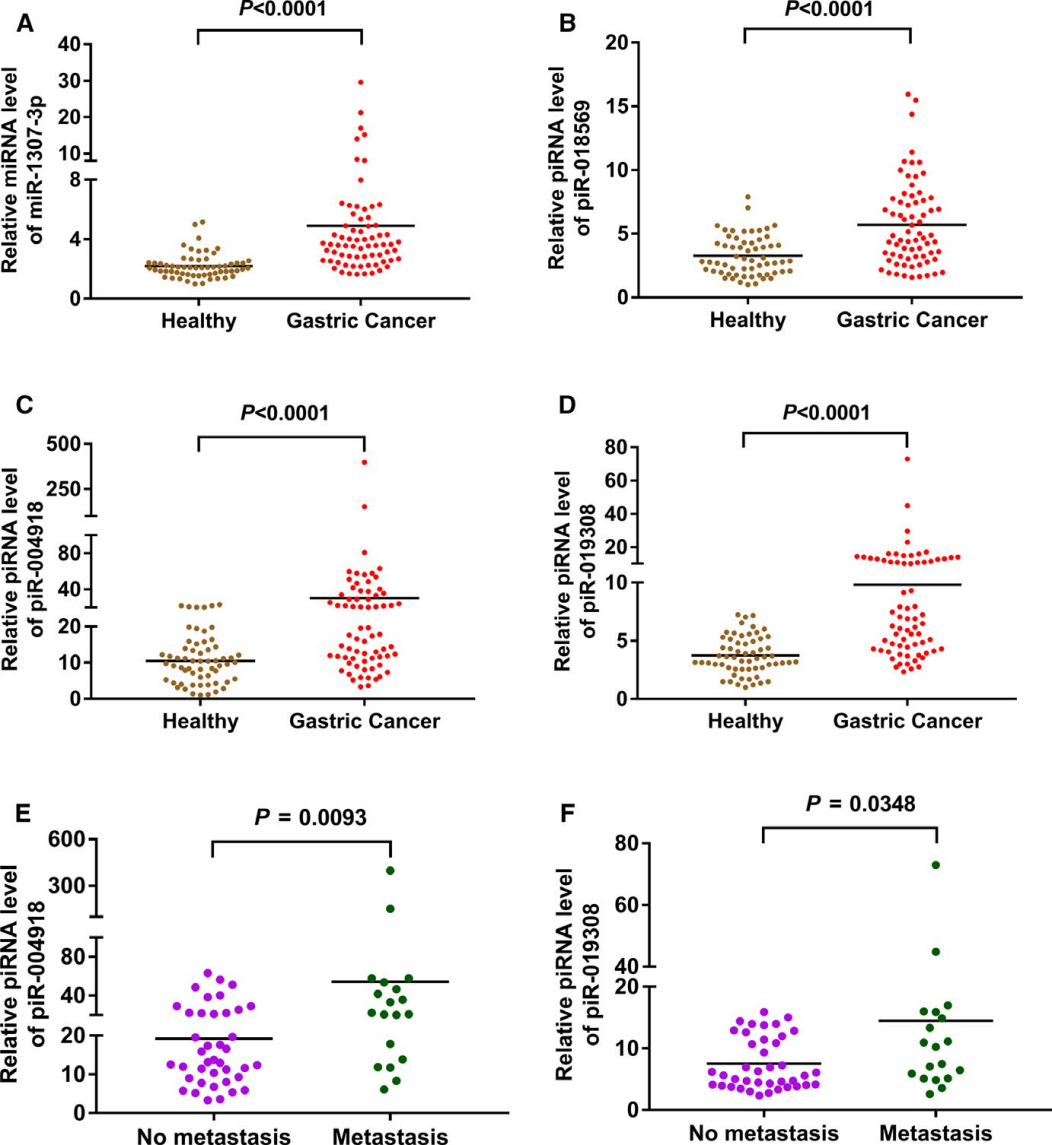
The expressions of miR‐1307‐3p, piR‐018569, piR‐004918 and piR‐019308 in validation cohorts. A, Relative miRNA level of miR‐1307‐3p in serum exosomes of GC patients (n = 70) and HC (n = 60) was investigated by qRT‐PCR; (B‐D) Relative piRNA levels of piR‐018569 (B), piR‐004918 (C) and piR‐019308 (D) in serum exosomes of GC patients (n = 70) and HC (n = 60) were investigated by qRT‐PCR; (E‐F) Relative piRNA levels of piR‐004918 (E) and piR‐019308 (F) in serum exosomes of GC patients with (n = 19) or without (n = 40) metastasis

The expressions of miR‐1307‐3p, piR‐018569, piR‐004918 and piR‐019308 in serum exosomes were further checked in remaining available samples in the validation cohorts. For the total samples in training cohorts and validation cohorts, both the expressions of miR‐1307‐3p, piR‐018569, piR‐004918 and piR‐019308 were significantly increased (*P* < .0001) in GC patients (n = 70) as compared to those in HC (n = 60) (Figure 4A‐D). Further, we analysed correlation between expression of miR‐1307‐3p/piR‐018569/piR‐004918/piR‐019308 with clinical characteristics of GC patients. Our data showed that GC patients with metastasis had significantly higher (*P* < .05) expression levels of piR‐004918 and piR‐019308 in serum exosomes than GC patients without metastasis (Figure 4E‐F). However, no significant differences were detected for miR‐1307‐3p/piR‐018569/piR‐004918/piR‐019308 in patients with other clinical characteristics such as sex, age or grade (Table S4).

### 3.5 Diagnostic values of miR‐1307‐3p, piR‐018569, piR‐004918 and piR‐019308 as biomarkers for GC patients

**Figure 5:**
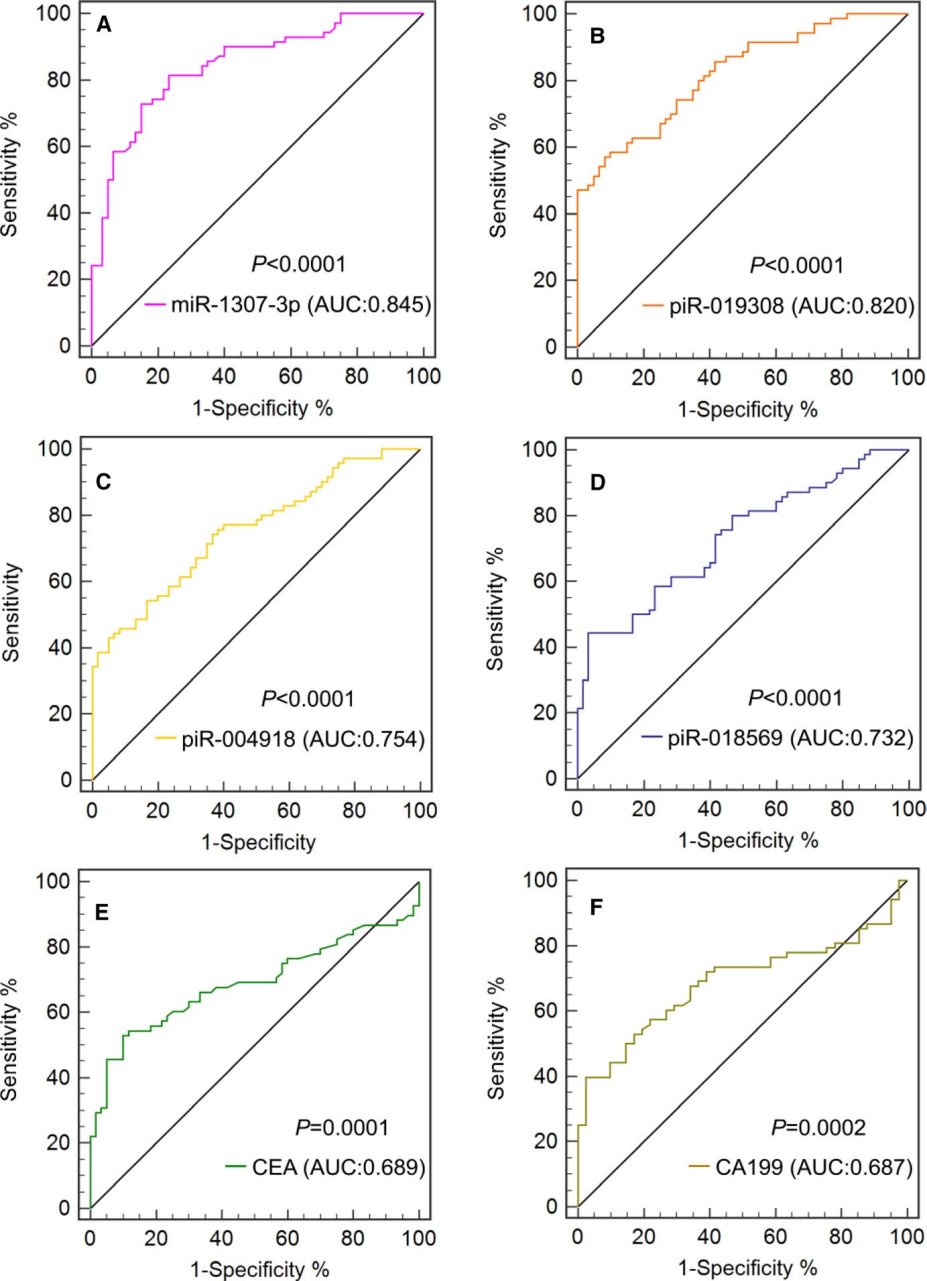
Clinical utility of serum exosomal miRNA, piRNAs, CEA and CA199 in the diagnosis of GC. The ROC curves for miR‐1307‐3p (A), piR‐019308 (B), piR‐004918 (C), piR‐018569 (D), CEA (E) and CA199 (F) including GC patients and HC

To test whether serum exosomal miR‐1307‐3p, piR‐018569, piR‐004918 and piR‐019308 levels can be used as diagnostic biomarkers for GC patients, the receiver operating characteristic (ROC) curve was plotted to identify a cut‐off value. As shown in Figure 5A‐D and Table S5, serum exosomal miR‐1307‐3p, piR‐019308, piR‐004918 and piR‐018569 levels could distinguish GC patients from HC, with an area under the curves (AUCs) of 0.845 (95% CI: 0.772 to 0.903, *P* < .0001), 0.820 (95% CI, 0.743‐0.882, *P* < .0001), 0.754 (95% CI, 0.671‐0.825, *P* < .0001) and 0.732 (95% CI, 0.647‐0.806, *P* < .0001), respectively. The results showed that the diagnostic values of miR‐1307‐3p, piR‐019308, piR‐004918 and piR‐018569 were much better than that of CEA, CA199 and AFP, which had the AUCs of 0.689, 0.687 and 0.634, respectively (Figure 5E‐F, Table S5).

### 3.6 Diagnostic values of miR‐1307‐3p/piR‐019308/piR‐004918/piR‐018569 combined with CEA and CA199

**Figure 6:**
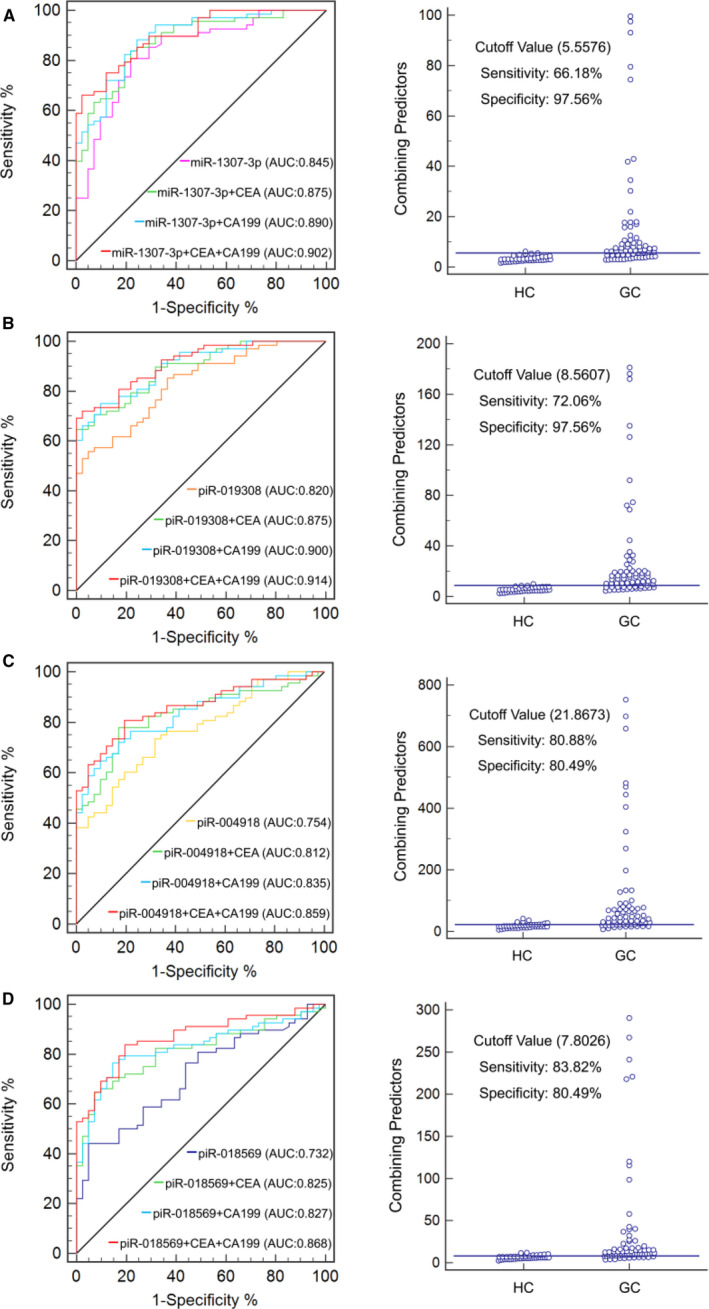
Clinical utility of serum exosomal miRNA or piRNAs combined with CEA and CA199 in the diagnosis of GC. The ROC curve (left) for miR‐1307‐3p (A), piR‐019308 (B), piR‐004918 (C) and piR‐018569 (D) combined with CEA or/and CA199 and cut‐off value (right) for miR‐1307‐3p/piR‐019308/piR‐004918/piR‐018569 CEA, CA199 in combination including GC patients and HC

When miR‐1307‐3p/piR‐019308/piR‐004918/piR‐018569 combined with CEA and CA199, the AUC of the biomarker panel reached 0.902 (95% CI, 0.830‐0.951, *P* < .0001), 0.914 (95% CI, 0.844‐0.959, *P* < .0001), 0.859 (95% CI, 0.780‐0.919, *P* < .0001) and 0.868 (95% CI, 0.790‐0.925, *P* < .0001), respectively. The optimal cut‐off values for miR‐1307‐3p/piR‐019308/piR‐004918/piR‐018569 combined with CEA and CA199 were 5.5576 (diagnostic sensitivity 66.18%, diagnostic specificity 97.56%), 8.5607 (diagnostic sensitivity 72.06%, diagnostic specificity 97.56%), 21.8673 (diagnostic sensitivity 80.88%, diagnostic specificity 80.49%) and 7.8026 (diagnostic sensitivity 83.82%, diagnostic specificity 80.49%), respectively (Figure 6). These data suggested that in addition to miR‐1307‐3p, serum exosomal piR‐019308 combined with CEA and CA199 could provide excellent diagnostic capabilities for GC patients.

### 3.7 Dysregulation of PIWI proteins expression in GC tissues

**Figure 7:**
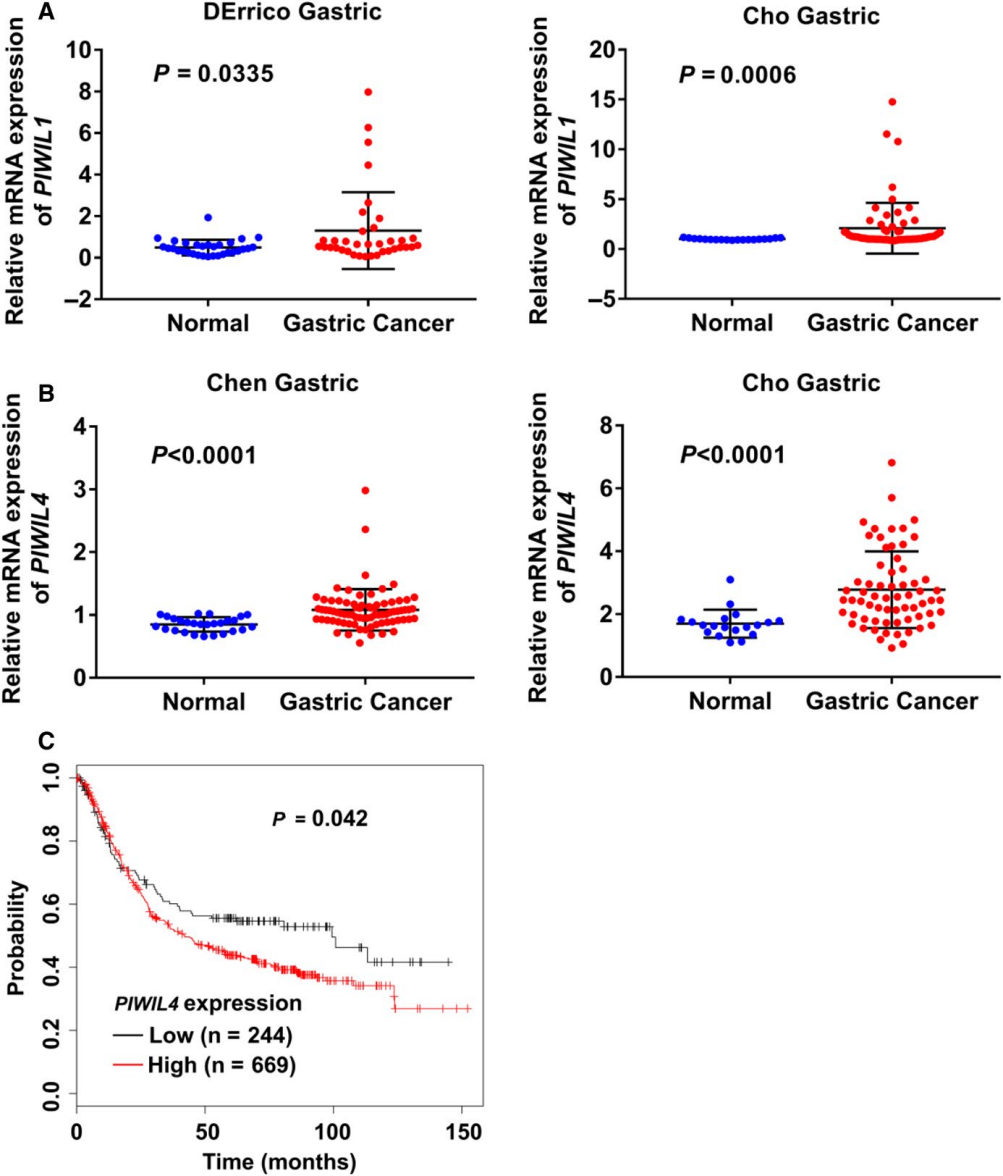
Expressions of*PIWIL1*,*PIWIL4*in GC tissues and the correlation between expression of*PIWIL4*and prognosis in GC patients. (A‐B) mRNA expressions of*PIWIL1*(A) and*PIWIL4*(B) in GC tissues compared with gastric normal tissues in two independent research projects from Oncomine database; (C) Correlation between expression of*PIWIL4*and OS (overall survival) in GC patients analysed by Kaplan‐Meier plotter

Accumulating studies revealed PIWI proteins might be frequently overexpressed in different cancer types.^33^, ^34^, ^35^ Through the Oncomine database (https://www.oncomine.org/), we found mRNA expressions of *PIWIL1* and *PIWIL4*, two of the major PIWI proteins, were significantly up‐regulated in GC tumour tissues compared with gastric normal mucosa tissues (Figure 7A‐B). To further explore the correlation between expressions of *PIWIL1*, *PIWIL4* and prognosis in GC patients, we analysed the overall survival (OS) by the use of Kaplan‐Meier plotter (https://kmplot.com/analysis/). We found *PIWIL4*‐high expression group significantly correlated with poor OS (Figure 7C). The above results indicated the overexpressed PIWI proteins might be involved in GC development and progression mediated by dysregulation of piRNAs. Tumour‐derived exosomes might package the piRNAs expressed abnormally in GC tissue, then released into the blood circulation.

## 4 DISCUSSION

The most commonly used GC tumour markers such as CEA, CA199, CA242 and CA724 are not diagnostically sensitive or specific enough to screen GC patients.^4^, ^5^ Thus, there is an urgent need to develop new and simple diagnostic biomarkers for detection of GC. In this study, we found that in addition to miR‐1307‐3p, serum exosomal piR‐019308, piR‐004918 and piR‐018569 could be novel biomarkers to screen gastric cancer. Furthermore, its combination with the current serum tumour markers such as CEA and CA199 can further enhance the diagnostic value of miR‐1307‐3p or piR‐019308 or piR‐004918 or piR‐018569.

Although accumulating studies have demonstrated dysregulation of miRNAs might be associated with various human cancers and used as promising biomarkers for cancers, the role of piRNAs in the development and progression of cancer is largely unknown, particularly the diagnostic value of piRNAs in serum exosomes for cancer patients has not been investigated. Our present study showed for the first time that in addition to miR‐1307‐3p, piR‐019308, piR‐004918 and piR‐018569 in serum exosomes were significantly increased in the GC group as compared to those in HC group. piRNAs could be more resistant to oxidation and degradation than miRNAs due to its 2’‐O‐Me modification on 3’ terminal base.^10^ Previous study reported piRNAs in serum or plasma remains extremely stable under repetitive freeze‐thaw cycles and a long time room‐temperature incubation.^36^ Moreover, the number of piRNAs has been identified more than 30,000 in human genome which is far greater than miRNAs (~2000).^12^ It indicated piRNAs could be potential and powerful biomarkers for cancer diagnosis and prognosis. One recent study has showed that serum piR‐020619 and piR‐020450 are promising novel biomarkers for early detection of colorectal cancer.^16^ Another study has revealed piR‐13643 and piR‐21238 could be novel diagnostic biomarkers for papillary thyroid carcinoma.^37^ In addition, piR‐10506469 were found to be significantly increased in the exosomes of plasma from both cholangiocarcinoma and gallbladder carcinoma patients, indicating it may be serve as the potential cancer biomarker.^38^ Exosomes secreted from tumour cells may contain several piRNAs originated from cells,^39^ which may be aberrantly expressed in tumour cells. Whether serum exosomal piRNAs are biomarkers for other cancers deserves further study.

The AUCs for miR‐1307‐3p, piR‐019308, piR‐004918 and piR‐018569 for discriminating GC patients from healthy controls were 0.845, 0.820, 0.754 and 0.732, respectively, which are markedly greater than that of CEA, CA199 and AFP. The combination of CEA and CA199 with miR‐1307‐3p/piR‐019308/ piR‐004918/piR‐018569 improved the AUCs to 0.902, 0.914, 0.859 and 0.868 respectively. These data suggested that in addition to miR‐1307‐3p, piR‐019308 combined with CEA and CA199 might be robust biomarkers for GC. Further, the expressions of piR‐004918 and piR‐019308 were significantly increased in the GC patients with metastasis as compared to patients without metastasis. It indicated that piR‐004918 and piR‐019308 might be potential markers for monitoring metastasis of GC.

PIWI proteins including PIWIL1, PIWIL2, PIWIL3 and PIWIL4 are critical for piRNA biogenesis.^9^ Accumulating studies revealed PIWI proteins might be frequently overexpressed in different cancer types including GC.^33^, ^34^, ^35^, ^40^ Through bioinformatics analysis, we found the expressions of *PIWIL1* and *PIWIL4* were increased in GC tumour tissues as compared to the normal gastric mucosa tissues whereas the high expression of *PIWIL4* in GC tumour tissues was correlation with poor OS in GC patients. It indicated that the overexpressed *PIWIL1* and *PIWIL4* might be involved in GC development and progression mediated by dysregulation of piRNA. Tumour‐derived exosomes might package the piRNAs expressed abnormally in GC tissues and then released into the blood circulation. Further work is needed to uncover the mechanisms responsible for the PIWI proteins‐induced dysregulation of piRNA and the correlation with the expression of piRNAs in GC tissues and serum exosomes.

## 5 CONCLUSION

In summary, our study showed for the first time that in addition to miR‐1307‐3p, serum exosomal piR‐019308, piR‐004918 and piR‐018569 are potential robust biomarkers for GC diagnosis. Moreover, serum exosomal piR‐004918 and piR‐019308 might be potential markers for monitoring metastasis of GC. The clinical values of piRNAs as biomarkers in other types of cancer need further investigation.

## CONFLICTS OF INTEREST

All authors declare that they have no competing interests.

## AUTHOR CONTRIBUTIONS

**Lichen Ge:** Conceptualization (lead); Data curation (lead); Formal analysis (lead); Methodology (lead); Writing‐original draft (lead). **Nan Zhang:** Data curation (equal); Formal analysis (supporting). **Dandan Li:** Data curation (supporting); Formal analysis (supporting). **Yingmin Wu:** Data curation (supporting); Formal analysis (supporting). **Hongsheng Wang:** Funding acquisition (equal); Project administration (equal); Supervision (equal); Writing‐review & editing (equal). **Junjun Wang:** Funding acquisition (lead); Supervision (lead); Writing‐review & editing (supporting).

## Supporting information

Table S1‐S5

## Data Availability

The data set used in this study is available from the corresponding authors on reasonable request.
