# Supplementary material for: Circulating exosomal small RNAs are promising non‐invasive diagnostic biomarkers for gastric cancer
Source: J Cell Mol Med. 2020 Nov 9;24(24):14502–13. doi: 10.1111/jcmm.16077 (PMC7753781; doi:10.1111/jcmm.16077)
Supplement: Supplementary file 1 — Table S1‐S5 [file JCMM-24-14502-s001.docx]

**Supplementary Table S1.** **Background demographic of the study cohorts.**

| **Parameter** | **HC** | **GC** | **Total** |
| --- | --- | --- | --- |
| **N** | 60 | 70 | 130 |
| **Age** |  |  |  |
| Mean ± SD | 59.0±9.2 | 59.4±11.2 | 59.2±10.3 |
| Range | 42-83 | 30-80 | 30-83 |
| **Sex** |  |  |  |
| Male | 40  (66.7%) | 40  (57.1%) | 80  (61.5%) |
| Female | 20  (33.3%) | 30  (42.9%) | 50  (38.5%) |

**Supplementary Table S2. Quantitative real-time PCR forward primers used in this study.**

| **Targets** | **Nucleotide sequence (5'->3')** |
| --- | --- |
| miR-140-5p | GCGCGCAGTGGTTTTACCCTATGGTAG |
| miR-660-5p | CGCGCTACCCATTGCATATCGGAGTTG |
| miR-1307-5p | TCGACCGGACCTCGACCGG |
| miR-20b-5p | CGCGCAAAGTGCTCATAGTGCAGGTAG |
| miR-378a-5p | CGCTCCTGACTCCAGGTCCTGTGT |
| miR-1307-3p | ACTCGGCGTGGCGTCGG |
| miR-12136 | CGCGCGAAAAAGTCATGGAGGCC |
| piR-018569 | CGCGCGTTGGTGGTTCAGTGGTAGAAT |
| piR-016945 | CGGCTAGCTCAGTCGGTAGAGCATGAG |
| piR-001918 | GCGCGTCAAACTCCTGACCTCATGATC |
| piR-004918 | TCGAACTCCTGACCTCAGGTGATCCG |
| piR-002433 | TCACTTCCCAGACGGAGTGGCTG |
| piR-019308 | GAGACCAGCCTGACCAACATGGTGGAA |
| piR-002528 | CGCGTCAGAGTGAACAGGCAACCTACA |
|  |  |

**Supplementary Table S3. The upregulated of miRNAs and piRNAs ranked top 10 in serum exosomal UMI small RNA sequencing of GC patients compared with HC.**

| **miRNAs** | **Fold change** | **piRNAs** | **Fold change** |
| --- | --- | --- | --- |
| miR-140-5p | 3.699 | piR-018569 | 2.652 |
| miR-660-5p | 2.286 | piR-016945 | 2.454 |
| miR-1307-5p | 2.146 | piR-001918 | 2.149 |
| miR-20b-5p | 2.103 | piR-004918 | 2.140 |
| miR-378a-5p | 2.057 | piR-002433 | 2.140 |
| miR-1307-3p | 1.951 | piR-020814 | 2.125 |
| miR-483-5p | 1.942 | piR-019308 | 2.123 |
| miR-3180 | 1.939 | piR-002528 | 2.105 |
| miR-12136 | 1.938 | piR-001101 | 2.041 |
| miR-1277-3p | 1.937 | piR-017138 | 2.037 |

**Supplementary Table S4. Relationship between the expression of miR-1307-3p, piR-019308, piR-004918, piR-018569 and the clinicopathological variables in GC patients.**

| **Variables** | **cases**  **(n)** | **Expression of**  **miR-1307-3p**  **(mean**±**SD)** | ***P*** | **Expression of**  **piR-019308**  **(mean±SD)** | ***P*** | **Expression of**  **piR-004918**  **(mean±SD)** | ***P*** | **Expression of**  **piR-018569**  **(mean±SD)** | ***P*** |
| --- | --- | --- | --- | --- | --- | --- | --- | --- | --- |
| **Sex** |  |  | 0.82 |  | 0.83 |  | 0.27 |  | 0.59 |
| Male | 40 | 4.658±3.465 |  | 9.703±11.32 |  | 30.25±61.61 |  | 5.664±2.889 |  |
| Female | 30 | 5.221±5.839 |  | 9.95±8.722 |  | 30.39±30.08 |  | 5.749±3.921 |  |
| **Age(year)** |  |  | 0.28 |  | 0.51 |  | 0.97 |  | 0.06 |
| <60 | 29 | 5.76±6.086 |  | 10.32±9.101 |  | 27.79±29.92 |  | 6.493±3.476 |  |
| ≥60 | 41 | 4.291±3.117 |  | 9.445±11.04 |  | 32.09±61.01 |  | 5.139±3.17 |  |
| **Grade** |  |  | 0.24 |  | 0.79 |  | 0.37 |  | 0.19 |
| G1+G2 | 18 | 5.147±4.055 |  | 8.263±4.713 |  | 23.4±16.55 |  | 4.715±2.743 |  |
| G3+G4 | 34 | 4.288±4.766 |  | 7.698±4.492 |  | 20.26±15.56 |  | 5.871±3.076 |  |
| **Stage** |  |  | 0.44 |  | 0.98 |  | 0.70 |  | 0.39 |
| Ⅰ+Ⅱ | 23 | 4.586±3.077 |  | 9.524±6.625 |  | 25.09±19.02 |  | 6.023±3.053 |  |
| Ⅲ+Ⅳ | 38 | 5.273±5.723 |  | 10.77±12.78 |  | 35.85±66.29 |  | 5.519±3.417 |  |

| **Stage** |  |  | 0.68 |  | 0.38 |  | 0.38 |  | 0.88 |
| --- | --- | --- | --- | --- | --- | --- | --- | --- | --- |
| Ⅰ | 13 | 4.279±1.319 |  | 9.45±7.148 |  | 26.83±22.01 |  | 5.668±3.302 |  |
| Ⅳ | 19 | 5.437±4.896 |  | 14.42±16.98 |  | 54±89.71 |  | 5.65±3.641 |  |
| **Stage** |  |  | 0.64 |  | 0.57 |  | 0.29 |  | 0.40 |
| Ⅱ | 10 | 4.986±4.528 |  | 9.62±6.257 |  | 22.83±15.12 |  | 6.484±2.798 |  |
| Ⅳ | 19 | 5.437±4.896 |  | 14.42±16.98 |  | 54±89.71 |  | 5.65±3.641 |  |
| **T status** |  |  | 0.20 |  | 0.78 |  | 0.66 |  | 0.50 |
| T1+T2 | 16 | 4.934±3.478 |  | 10.16±7.419 |  | 27.74±22.12 |  | 5.968±3.065 |  |
| T3+T4 | 45 | 5.042±5.32 |  | 10.35±11.88 |  | 33.23±61.15 |  | 5.617±3.366 |  |
| **N status** |  |  | 0.59 |  | 0.87 |  | 0.39 |  | 0.39 |
| No | 20 | 3.894±1.585 |  | 8.807±6.371 |  | 23.25±18.94 |  | 6.051±3.215 |  |
| Yes | 22 | 5.461±6.635 |  | 8.039±5.383 |  | 20.15±18.22 |  | 5.233±2.948 |  |
| **Metastasis** |  |  | 0.11 |  | **0.0348** |  | **0.0093** |  | 0.92 |
| No | 40 | 4.713±5.508 |  | 7.514±4.228 |  | 19.18±14.91 |  | 5.524±3.082 |  |
| Yes | 19 | 5.675±4.812 |  | 14.48±16.94 |  | 54.27±89.57 |  | 5.9±3.756 |  |
| **CEA (ng/ml)** |  |  | 0.99 |  | 0.91 |  | 0.38 |  | 0.19 |
| <5 | 47 | 4.912±4.776 |  | 9.845±10.93 |  | 29.34±57.39 |  | 6.011±3.354 |  |
| ≥5 | 21 | 4.87±4.547 |  | 9.551±9.115 |  | 31.67±33.01 |  | 5.081±3.339 |  |

| **CA199 (IU/ml)** |  |  | 0.64 |  | 0.45 |  | 0.97 |  | >0.99 |
| --- | --- | --- | --- | --- | --- | --- | --- | --- | --- |
| <37 | 51 | 4.984±4.755 |  | 9.989±10.57 |  | 31.13±55.64 |  | 5.708±3.327 |  |
| ≥37 | 17 | 4.646±4.546 |  | 9.05±9.886 |  | 26.83±33.9 |  | 5.771±3.531 |  |
| **AFP (ng/ml )** |  |  | 0.83 |  | 0.91 |  | 0.87 |  | 0.13 |
| <20 | 62 | 5.05±4.841 |  | 10.08±10.72 |  | 30.75±52.86 |  | 5.923±3.414 |  |
| ≥20 | 4 | 4.048±1.928 |  | 7.926±3.693 |  | 30.89±25.05 |  | 3.559±1.95 |  |
| **CA242 (IU/ml)** |  |  | 0.68 |  | 0.66 |  | 0.92 |  | 0.86 |
| <20 | 48 | 4.818±4.666 |  | 10.2±10.82 |  | 31.66±57.23 |  | 5.431±3.029 |  |
| ≥20 | 11 | 3.862±1.84 |  | 7.403±3.595 |  | 21.05±11.21 |  | 5.536±2.706 |  |
| **CA724 (IU/ml)** |  |  | 0.97 |  | 0.81 |  | 0.78 |  | 0.82 |
| <6 | 46 | 4.508±3.271 |  | 9.753±10.61 |  | 30.96±57.67 |  | 5.238±2.693 |  |
| ≥6 | 15 | 5.552±6.896 |  | 9.156±7.239 |  | 24.8±22.17 |  | 5.754±3.551 |  |
| **CA50 (IU/ml)** |  |  | 0.58 |  | 0.53 |  | 0.54 |  | 0.45 |
| <25 | 48 | 4.989±4.811 |  | 10.02±10.75 |  | 32.92±56.95 |  | 5.216±2.991 |  |
| ≥25 | 10 | 3.869±1.953 |  | 7.404±4.252 |  | 19.85±12.06 |  | 5.779±2.576 |  |
| **CA125 (IU/ml)** |  |  | 0.71 |  | 0.70 |  | 0.82 |  | 0.38 |
| <35 | 54 | 4.783±4.618 |  | 9.542±10.33 |  | 29.98±54.1 |  | 5.422±2.98 |  |
| ≥35 | 14 | 5.349±5.028 |  | 10.57±10.73 |  | 30.37±37.5 |  | 6.89±4.458 |  |

The GC stage was assessed by the TNM system according to the *American Joint Committee on Cancer Staging Manual, Seventh Edition*.

**Supplementary Table S5. Clinical diagnosis utility about various marker alone and their combination test, for GC patients and HC.**

|  | **HC vs GC** | | | | |
| --- | --- | --- | --- | --- | --- |
| **Marker** | **AUC** | **Sensitivity%** | **Specificity%** | ***P* value** | **95% CI** |
| **miR-1307-3p** | 0.845 | 81.43 | 76.67 | <0.0001 | 0.772-0.903 |
| **piR-019308** | 0.820 | 57.14 | 91.67 | <0.0001 | 0.743-0.882 |
| **piR-004918** | 0.754 | 42.86 | 95.00 | <0.0001 | 0.671-0.825 |
| **piR-018569** | 0.732 | 44.29 | 96.67 | <0.0001 | 0.647-0.806 |
| **CEA** | 0.689 | 52.94 | 90.00 | 0.0001 | 0.601-0.768 |
| **CA199** | 0.687 | 39.71 | 97.56 | 0.0002 | 0.591-0.773 |
| **AFP** | 0.634 | 57.58 | 66.67 | 0.0075 | 0.543-0.718 |
| **miR-1307-3p+CEA** | 0.875 | 83.82 | 78.33 | <0.0001 | 0.804-0.926 |
| **miR-1307-3p+CA199** | 0.890 | 88.24 | 75.61 | <0.0001 | 0.816-0.942 |
| **miR-1307-3p**  **+CEA+CA199** | 0.902 | 66.18 | 97.56 | <0.0001 | 0.830-0.951 |
| **piR-019308+CEA** | 0.875 | 64.71 | 95.00 | <0.0001 | 0.805-0.927 |
| **piR-019308+CA199** | 0.900 | 75.00 | 90.24 | <0.0001 | 0.828-0.949 |
| **piR-019308**  **+CEA+CA199** | 0.914 | 72.06 | 97.56 | <0.0001 | 0.844-0.959 |
| **piR-004918+CEA** | 0.812 | 77.94 | 78.33 | <0.0001 | 0.733- 0.876 |
| **piR-004918+CA199** | 0.835 | 72.06 | 82.93 | <0.0001 | 0.751- 0.899 |
| **piR-004918** | 0.859 | 80.88 | 80.49 | <0.0001 | 0.780-0.919 |
| **+CEA+CA199** |  |  |  |  |  |
| **piR-018569+CEA** | 0.825 | 64.71 | 95.00 | <0.0001 | 0.748-0.886 |
| **piR-018569+CA199** | 0.827 | 76.47 | 85.37 | <0.0001 | 0.743-0.893 |
| **piR-018569**  **+CEA+CA199** | 0.868 | 83.82 | 80.49 | <0.0001 | 0.790-0.925 |
